# Indiana

**Published:** 1896-09

**Authors:** 


*Indiana.* At Fort Wayne, a dairyman has sued the State Board of Health for two thousand dollars damages, basing his claim on the condemnation and killing of five cows afflicted with tuberculosis. He claims that a reputable physician and veterinarian on examination pronounced these animals in perfect condition and health. It will be interesting to watch the outcome of this case.

